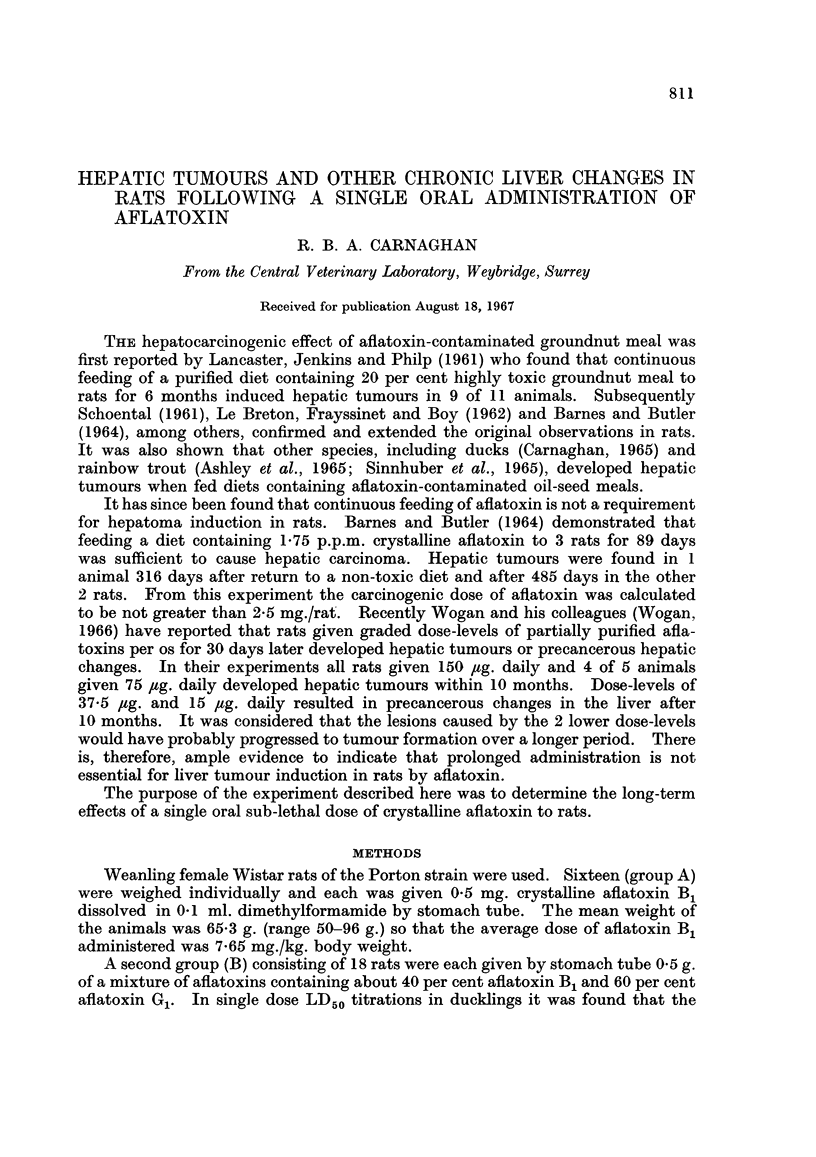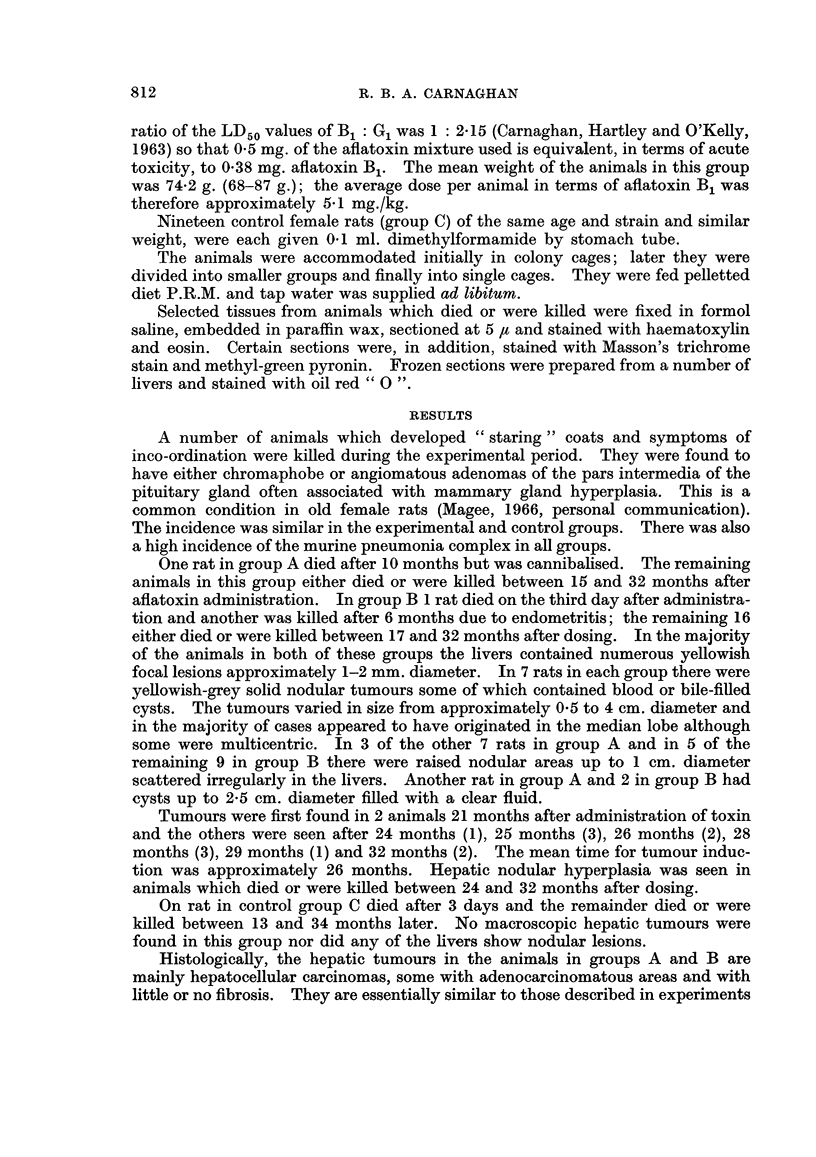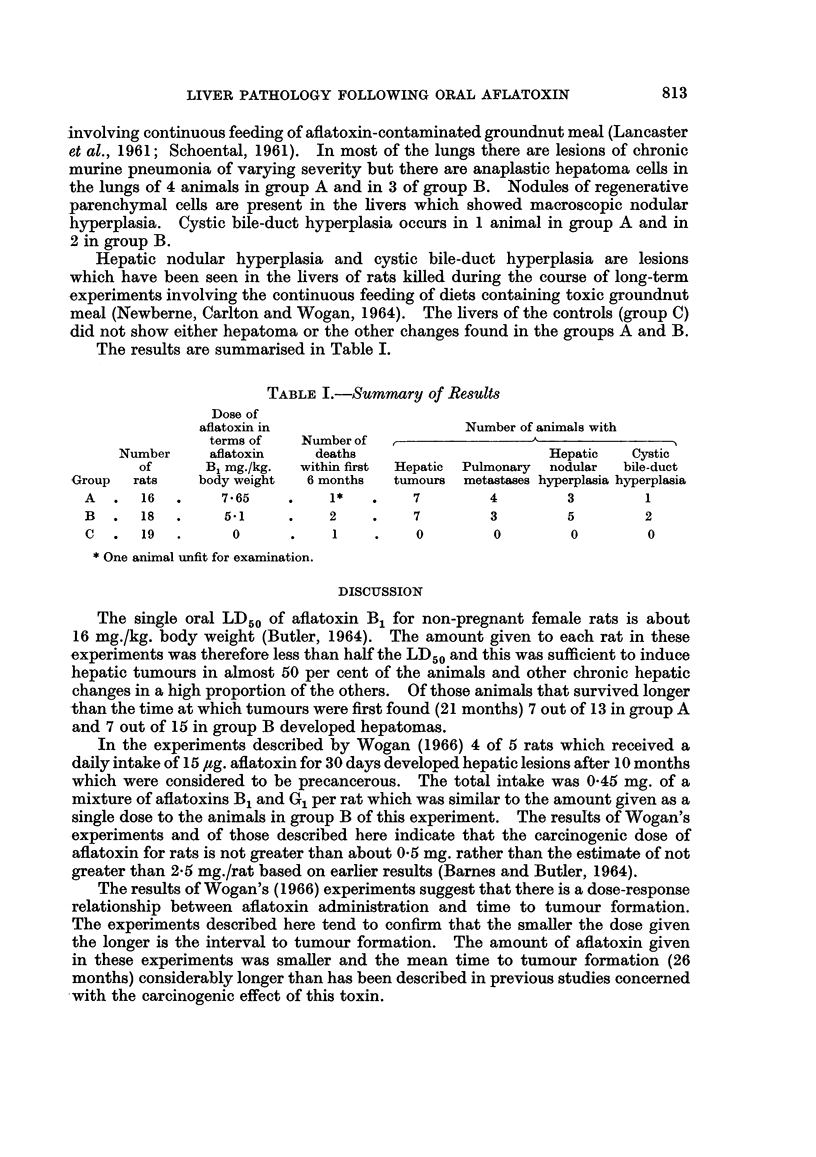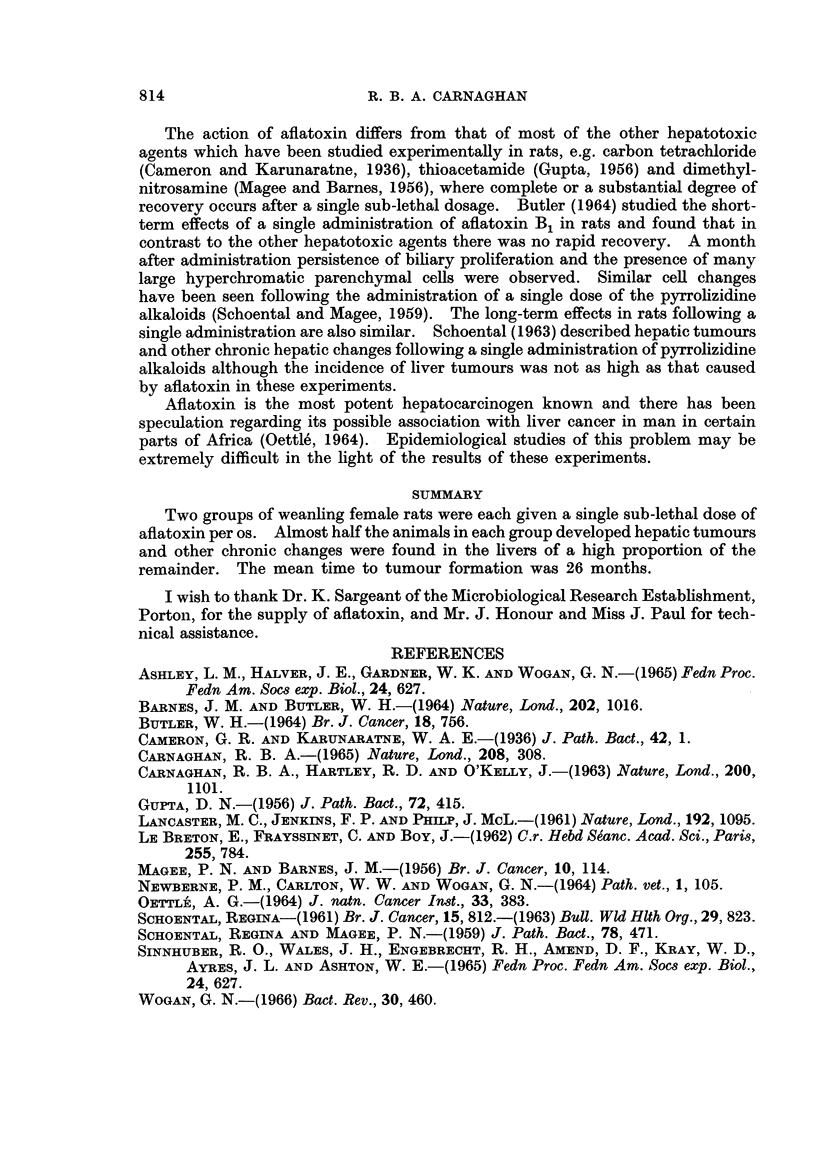# Hepatic tumours and other chronic liver changes in rats following a single oral administration of aflatoxin.

**DOI:** 10.1038/bjc.1967.95

**Published:** 1967-12

**Authors:** R. B. Carnaghan


### HEPATIC TUMOURS AND OTHER CHRONIC LIVER CHANGES IN RATS FOLLOWING A SINGLE ORAL ADMINISTRATION OF AFLATOXIN

R. B. A. CARNAGHAN

*From the Central Veterinary Laboratory, Weybridge, Surrey*

Received for publication August 18, 1967

THE hepatocarcinogenic effect of aflatoxin-contaminated groundnut meal was first reported by Lancaster, Jenkins and Philp (1961) who found that continuous feeding of a purified diet containing 20 per cent highly toxic groundnut meal to rats for 6 months induced hepatic tumours in 9 of 11 animals. Subsequently Schoental (1961), Le Breton, Frayssinet and Boy (1962) and Barnes and Butler (1964), among others, confirmed and extended the original observations in rats. It was also shown that other species, including ducks (Carnaghan, 1965) and rainbow trout (Ashley *et al.*, 1965; Sinnhuber *et al.*, 1965), developed hepatic tumours when fed diets containing aflatoxin-contaminated oil-seed meals.

It has since been found that continuous feeding of aflatoxin is not a requirement for hepatoma induction in rats. Barnes and Butler (1964) demonstrated that feeding a diet containing 1·75 p.p.m. crystalline aflatoxin to 3 rats for 89 days was sufficient to cause hepatic carcinoma. Hepatic tumours were found in 1 animal 316 days after return to a non-toxic diet and after 485 days in the other 2 rats. From this experiment the carcinogenic dose of aflatoxin was calculated to be not greater than 2·5 mg./rat. Recently Wogan and his colleagues (Wogan, 1966) have reported that rats given graded dose-levels of partially purified aflatoxins per os for 30 days later developed hepatic tumours or precancerous hepatic changes. In their experiments all rats given 150 $\mu$g. daily and 4 of 5 animals given 75 $\mu$g. daily developed hepatic tumours within 10 months. Dose-levels of 37·5 $\mu$g. and 15 $\mu$g. daily resulted in precancerous changes in the liver after 10 months. It was considered that the lesions caused by the 2 lower dose-levels would have probably progressed to tumour formation over a longer period. There is, therefore, ample evidence to indicate that prolonged administration is not essential for liver tumour induction in rats by aflatoxin.

The purpose of the experiment described here was to determine the long-term effects of a single oral sub-lethal dose of crystalline aflatoxin to rats.

### METHODS

Weanling female Wistar rats of the Porton strain were used. Sixteen (group A) were weighed individually and each was given 0·5 mg. crystalline aflatoxin $B_1$ dissolved in 0·1 ml. dimethylformamide by stomach tube. The mean weight of the animals was 65·3 g. (range 50–96 g.) so that the average dose of aflatoxin $B_1$ administered was 7·65 mg./kg. body weight.

A second group (B) consisting of 18 rats were each given by stomach tube 0·5 g. of a mixture of aflatoxins containing about 40 per cent aflatoxin $B_1$ and 60 per cent aflatoxin $G_1$. In single dose $LD_{50}$ titrations in ducklings it was found that the ratio of the $LD_{50}$ values of $B_1$ : $G_1$ was 1 : 2·15 (Carnaghan, Hartley and O'Kelly, 1963) so that 0·5 mg. of the aflatoxin mixture used is equivalent, in terms of acute toxicity, to 0·38 mg. aflatoxin $B_1$. The mean weight of the animals in this group was 74·2 g. (68–87 g.); the average dose per animal in terms of aflatoxin $B_1$ was therefore approximately 5·1 mg./kg.

Nineteen control female rats (group C) of the same age and strain and similar weight, were each given 0·1 ml. dimethylformamide by stomach tube.

The animals were accommodated initially in colony cages; later they were divided into smaller groups and finally into single cages. They were fed pelletted diet P.R.M. and tap water was supplied *ad libitum.*

Selected tissues from animals which died or were killed were fixed in formol saline, embedded in paraffin wax, sectioned at 5 $\mu$ and stained with haematoxylin and eosin. Certain sections were, in addition, stained with Masson's trichrome stain and methyl-green pyronin. Frozen sections were prepared from a number of livers and stained with oil red " O ".

### RESULTS

A number of animals which developed " staring " coats and symptoms of inco-ordination were killed during the experimental period. They were found to have either chromaphobe or angiomatous adenomas of the pars intermedia of the pituitary gland often associated with mammary gland hyperplasia. This is a common condition in old female rats (Magee, 1966, personal communication). The incidence was similar in the experimental and control groups. There was also a high incidence of the murine pneumonia complex in all groups.

One rat in group A died after 10 months but was cannibalised. The remaining animals in this group either died or were killed between 15 and 32 months after aflatoxin administration. In group B 1 rat died on the third day after administration and another was killed after 6 months due to endometritis; the remaining 16 either died or were killed between 17 and 32 months after dosing. In the majority of the animals in both of these groups the livers contained numerous yellowish focal lesions approximately 1–2 mm. diameter. In 7 rats in each group there were yellowish-grey solid nodular tumours some of which contained blood or bile-filled cysts. The tumours varied in size from approximately 0·5 to 4 cm. diameter and in the majority of cases appeared to have originated in the median lobe although some were multicentric. In 3 of the other 7 rats in group A and in 5 of the remaining 9 in group B there were raised nodular areas up to 1 cm. diameter scattered irregularly in the livers. Another rat in group A and 2 in group B had cysts up to 2·5 cm. diameter filled with a clear fluid.

Tumours were first found in 2 animals 21 months after administration of toxin and the others were seen after 24 months (1), 25 months (3), 26 months (2), 28 months (3), 29 months (1) and 32 months (2). The mean time for tumour induction was approximately 26 months. Hepatic nodular hyperplasia was seen in animals which died or were killed between 24 and 32 months after dosing.

On rat in control group C died after 3 days and the remainder died or were killed between 13 and 34 months later. No macroscopic hepatic tumours were found in this group nor did any of the livers show nodular lesions.

Histologically, the hepatic tumours in the animals in groups A and B are mainly hepatocellular carcinomas, some with adenocarcinomatous areas and with little or no fibrosis. They are essentially similar to those described in experiments involving continuous feeding of aflatoxin-contaminated groundnut meal (Lancaster *et al.*, 1961; Schoental, 1961). In most of the lungs there are lesions of chronic murine pneumonia of varying severity but there are anaplastic hepatoma cells in the lungs of 4 animals in group A and in 3 of group B. Nodules of regenerative parenchymal cells are present in the livers which showed macroscopic nodular hyperplasia. Cystic bile-duct hyperplasia occurs in 1 animal in group A and in 2 in group B.

Hepatic nodular hyperplasia and cystic bile-duct hyperplasia are lesions which have been seen in the livers of rats killed during the course of long-term experiments involving the continuous feeding of diets containing toxic groundnut meal (Newberne, Carlton and Wogan, 1964). The livers of the controls (group C) did not show either hepatoma or the other changes found in the groups A and B.

The results are summarised in Table I.

TABLE I.—*Summary of Results*

| Group | Number of rats | Dose of aflatoxin in terms of aflatoxin $B_1$ mg./kg. body weight | Number of deaths within first 6 months | Number of animals with: Hepatic tumours | Pulmonary metastases | Hepatic nodular hyperplasia | Cystic bile-duct hyperplasia |
|---|---|---|---|---|---|---|---|
| A | 16 | 7·65 | 1* | 7 | 4 | 3 | 1 |
| B | 18 | 5·1 | 2 | 7 | 3 | 5 | 2 |
| C | 19 | 0 | 1 | 0 | 0 | 0 | 0 |

* One animal unfit for examination.

### DISCUSSION

The single oral $LD_{50}$ of aflatoxin $B_1$ for non-pregnant female rats is about 16 mg./kg. body weight (Butler, 1964). The amount given to each rat in these experiments was therefore less than half the $LD_{50}$ and this was sufficient to induce hepatic tumours in almost 50 per cent of the animals and other chronic hepatic changes in a high proportion of the others. Of those animals that survived longer than the time at which tumours were first found (21 months) 7 out of 13 in group A and 7 out of 15 in group B developed hepatomas.

In the experiments described by Wogan (1966) 4 of 5 rats which received a daily intake of 15 $\mu$g. aflatoxin for 30 days developed hepatic lesions after 10 months which were considered to be precancerous. The total intake was 0·45 mg. of a mixture of aflatoxins $B_1$ and $G_1$ per rat which was similar to the amount given as a single dose to the animals in group B of this experiment. The results of Wogan's experiments and of those described here indicate that the carcinogenic dose of aflatoxin for rats is not greater than about 0·5 mg. rather than the estimate of not greater than 2·5 mg./rat based on earlier results (Barnes and Butler, 1964).

The results of Wogan's (1966) experiments suggest that there is a dose-response relationship between aflatoxin administration and time to tumour formation. The experiments described here tend to confirm that the smaller the dose given the longer is the interval to tumour formation. The amount of aflatoxin given in these experiments was smaller and the mean time to tumour formation (26 months) considerably longer than has been described in previous studies concerned with the carcinogenic effect of this toxin.

The action of aflatoxin differs from that of most of the other hepatotoxic agents which have been studied experimentally in rats, e.g. carbon tetrachloride (Cameron and Karunaratne, 1936), thioacetamide (Gupta, 1956) and dimethylnitrosamine (Magee and Barnes, 1956), where complete or a substantial degree of recovery occurs after a single sub-lethal dosage. Butler (1964) studied the short-term effects of a single administration of aflatoxin $B_1$ in rats and found that in contrast to the other hepatotoxic agents there was no rapid recovery. A month after administration persistence of biliary proliferation and the presence of many large hyperchromatic parenchymal cells were observed. Similar cell changes have been seen following the administration of a single dose of the pyrrolizidine alkaloids (Schoental and Magee, 1959). The long-term effects in rats following a single administration are also similar. Schoental (1963) described hepatic tumours and other chronic hepatic changes following a single administration of pyrrolizidine alkaloids although the incidence of liver tumours was not as high as that caused by aflatoxin in these experiments.

Aflatoxin is the most potent hepatocarcinogen known and there has been speculation regarding its possible association with liver cancer in man in certain parts of Africa (Oettlé, 1964). Epidemiological studies of this problem may be extremely difficult in the light of the results of these experiments.

SUMMARY

Two groups of weanling female rats were each given a single sub-lethal dose of aflatoxin per os. Almost half the animals in each group developed hepatic tumours and other chronic changes were found in the livers of a high proportion of the remainder. The mean time to tumour formation was 26 months.

I wish to thank Dr. K. Sargeant of the Microbiological Research Establishment, Porton, for the supply of aflatoxin, and Mr. J. Honour and Miss J. Paul for technical assistance.

REFERENCES

Ashley, L. M., Halver, J. E., Gardner, W. K. and Wogan, G. N.—(1965) *Fedn Proc. Fedn Am. Socs exp. Biol.*, **24**, 627.
Barnes, J. M. and Butler, W. H.—(1964) *Nature, Lond.*, **202**, 1016.
Butler, W. H.—(1964) *Br. J. Cancer*, **18**, 756.
Cameron, G. R. and Karunaratne, W. A. E.—(1936) *J. Path. Bact.*, **42**, 1.
Carnaghan, R. B. A.—(1965) *Nature, Lond.*, **208**, 308.
Carnaghan, R. B. A., Hartley, R. D. and O'Kelly, J.—(1963) *Nature, Lond.*, **200**, 1101.
Gupta, D. N.—(1956) *J. Path. Bact.*, **72**, 415.
Lancaster, M. C., Jenkins, F. P. and Philp, J. McL.—(1961) *Nature, Lond.*, **192**, 1095.
Le Breton, E., Frayssinet, C. and Boy, J.—(1962) *C.r. Hebd Séanc. Acad. Sci., Paris*, **255**, 784.
Magee, P. N. and Barnes, J. M.—(1956) *Br. J. Cancer*, **10**, 114.
Newberne, P. M., Carlton, W. W. and Wogan, G. N.—(1964) *Path. vet.*, **1**, 105.
Oettlé, A. G.—(1964) *J. natn. Cancer Inst.*, **33**, 383.
Schoental, Regina—(1961) *Br. J. Cancer*, **15**, 812.—(1963) *Bull. Wld Hlth Org.*, **29**, 823.
Schoental, Regina and Magee, P. N.—(1959) *J. Path. Bact.*, **78**, 471.
Sinnhuber, R. O., Wales, J. H., Engebrecht, R. H., Amend, D. F., Kray, W. D., Ayres, J. L. and Ashton, W. E.—(1965) *Fedn Proc. Fedn Am. Socs exp. Biol.*, **24**, 627.
Wogan, G. N.—(1966) *Bact. Rev.*, **30**, 460.